# The Positive Effect of *Akkermansia muciniphila* postbiotics on the Glycolipid Metabolism of *Caenorhabditis elegans* Induced by High-Glucose Diet

**DOI:** 10.3390/nu17060976

**Published:** 2025-03-11

**Authors:** Zhongqin Wu, Ke Li, Aixing Hou, Yuanliang Wang, Zongjun Li

**Affiliations:** 1 Hunan Province Key Laboratory of Food Science and Biotechnology, College of Food Science and Technology, Hunan Agricultural University, Changsha 410128, China; hnwuzhongqin@163.com (Z.W.); wangyuanliang@hunau.edu.cn (Y.W.); 2 School of Pharmaceutical and Bioengineering, Hunan Chemical Vocational Technology College, Zhuzhou 412000, China; 3 National Research Center of Engineering Technology for Utilization of Functional Ingredients from Botanicals, Changsha 410128, China

**Keywords:** *Akkermansia muciniphila*, postbiotics, *Caenorhabditis elegans*, healthy lifespan, glucolipid metabolism, high-glucose diet

## Abstract

Background: Glycolipid metabolism is essential for maintaining metabolic homeostasis. As a new postbiotic, pasteurized *Akkermansia muciniphila* (P-AKK) is important for the regulation of immunity and metabolism. Objectives: This study aimed to evaluate the effects of P-AKK on glycolipid metabolism in *Caenorhabditis elegans* fed a high glucose diet. Results: We discovered that feeding nematodes P-AKK improved their healthy lifespan when fed a high-glucose diet. Furthermore, P-AKK contributes to reducing the accumulation of glucose, advanced glycation end products, and lipids and maintains a better physiological state. In addition, P-AKK improved the composition of free fatty acids and decreased the total free fatty acid content of *C. elegans*. Transcriptome sequencing analysis revealed that P-AKK induced significant enrichment of carbohydrate, oxidative phosphorylation, and fatty acid metabolism pathways. These significantly enriched biological processes were closely related to glucose and lipid metabolism. Among them, P-AKK activated the β-oxidation of fatty acids while inhibiting the de novo synthesis of fatty acids to regulate fatty acid metabolism. Conclusions: The administration of P-AKK positively affected the body phenotypes of *C. elegans* under high glucose conditions. P-AKK mitigated the fat accumulation induced by a high-glucose diet by regulating key metabolic enzymes, including acyl-CoA synthetase and stearoyl-CoA desaturase.

## 1. Introduction

In recent years, Western eating habits, which are rich in meat and refined sugar, have gradually begun to prevail in China. The narrative must shift away from individual choice to structural factors, as more than half of the global population will be living with overweight or obesity by 2035 if current trends continue, and more than 1.3 billion people worldwide will have diabetes by 2050 [1]. Overconsumption of sugar causes most new cases, fueled by changes in obesity and dietary risk. Therefore, we must pay more attention to diet and health.

The intestine plays a vital role in nutrient digestion and absorption, especially by gut microorganisms. Moreover, the intestinal microbiota is susceptible to dietary nutrients, which subsequently influence metabolic levels in a beneficial manner [2,3]. A study reported that a modified dietary structure has a positive effect on lipid metabolism in obese mice because the abundance of *Akkermansia, Lactobacillus*, and other beneficial microbes increases in the gut [4]. *Akkermansia muciniphila* is a promising probiotic candidate that promotes metabolic balance. Compared with individuals with normal glucose tolerance, the abundance of *Akkermansia muciniphila* in the fecal samples of patients with prediabetes was lower, suggesting that there may be a negative correlation between them [5]. In addition, because diabetes and obesity are related to intestinal permeability and low-grade inflammation, *Akkermansia muciniphila* inhibits the occurrence of disease by promoting intestinal microbial diversity and intestinal health by improving healthy mucous layer circulation [6,7]. Even *Akkermansia muciniphila* is expected to become the next generation of probiotics, similar to *Lactobacillus* and *Bifidobacteria*. Dietary probiotic interventions are currently popular means of regulating dietary patterns. For example, oral administration of *Lactobacillus plantarum* Q16 inhibited metabolic disorders of the gut microbiota and improved fat and energy metabolism in mice fed a high-fat diet [8]. Plovier et al. first showed that pasteurized *Akkermansia muciniphila* (P-AKK) augments its beneficial effects on obesity and insulin resistance in HFD-fed mice and that Amuc_1100, a thermostable outer-membrane protein of *A. muciniphila*, recapitulates most of these effects [9,10]. This suggests that *A. muciniphila* retains its biological activity at pasteurization temperatures and exerts beneficial effects. However, it is unclear how P-AKK, as a novel postbiotic, regulates host physiological pathways such as fat distribution and energy homeostasis.

*Caenorhabditis elegans* is a model organism that feeds on *Escherichia coli* OP50. Bacteria provide essential nutrients for the growth and development of nematodes. Therefore, the *Caenorhabditis elegans* model allows direct changes in the bacterial diet to be used to study its effects on host physiology. In this study, *Caenorhabditis elegans* fed a high-glucose diet was used to investigate the effects of P-AKK on the glycolipid metabolism pathways. Accordingly, we tested the lifespan, glycolipid metabolism-related markers, and behavioral phenotypes of *C. elegans*. To further elucidate the positive effects of P-AKK on glycolipid metabolism, we conducted RNA sequencing and quantitative real-time polymerase chain reaction (PCR) analysis of differentially expressed genes (DEGs) in *C. elegans* induced by P-AKK.

## 2. Materials and Methods

### 2.1. Bacterial Culture Conditions and Strains

*Akkermansia muciniphila* (ATCC BAA-835) was obtained from the American Type Culture Collection (ATCC). The culture and enrichment of *A. muciniphila* and *Escherichia coli* OP50 were performed as described previously [11]. P-AKK was prepared as described previously [12]. Briefly, the *A. muciniphila* concentrate was pasteurized for 30 min at 70 °C. It was then immediately stored at −80 °C until use.

*Caenorhabditis elegans* and *Escherichia coli* OP50 were originally obtained from the Caenorhabditis Genetics Center (University of Minnesota, MN, USA). N2 Bristol was used as the wild-type strain. *Escherichia coli* OP50 was used as a standard food for *C. elegans*. P-AKK was used as a test food source for *C. elegans*. To explore the physiological changes caused by nematodes under different dietary patterns, nematodes were divided into OP50 and OP50 with 2% glucose, P-AKK, and P-AKK with 2% glucose groups. *C. elegans* were synchronized and normally fed to the L4 stage and then treated with *E. coli* OP50 or P-AKK for 24 h to reach adulthood on nematode growth medium plates with or without glucose.

A high-glucose diet culture plate [13]: Peptone-free modified nematode growth medium (mNGM) was autoclaved at 121 °C for 20 min and then cooled in a 55 °C water bath for 15 min. Glucose water (the final concentration of glucose was 2%) and FUdR (final concentration of 0.12 mM) were added before pouring.

Normal diet culture plates were arranged according to the standardized mNGM protocol without the addition of additional glucose.

### 2.2. Lifespan Assay

Worms at the L4 stage were transferred to the corresponding treatment plate, and this time was denoted as day 0. For the first seven days, the worms were transferred to fresh mNGM every day, after which they were transferred once per week for the remainder of the experiment to maintain a sufficient food source. Worms were scored daily by strong light exposure or mechanical stimulation with a platinum wire until all animals died. Worms that did not respond were scored as dead. The experiments were performed in triplicate with 20 ± 5 nematodes per plate. Statistical analysis of lifespan data was performed using Kaplan–Meier analysis.

### 2.3. Healthy Lifespan: Body Bends/Movements on mNGM Plates

L4 late larvae were transplanted into experimental plates and cultured for 24 h. At this time, the nematodes were 4 days old. Body bend frequency and movement status of 4-, 6-, 8-, 10-, and 12-day-old nematodes were determined.

The body bends and movement of *C. elegans* were defined according to previous methods [14,15].

### 2.4. Biochemical Assay

For each group, worms were washed from the plates with M9 buffer and transferred to fresh tubes. The worms were then homogenized using an ultrasonic cell crusher. After centrifugation at 8000× *g* for 10 min, the supernatants were used for subsequent assays.

Advanced glycosylation end product (AGE) levels were measured according to the manufacturer’s instructions (Shanghai Zcibio Technology Co., Ltd., Shanghai, China). Glucose and triglyceride (TG) contents were measured according to the manufacturer’s instructions (Solarbio Science and Technology Co., Ltd., Beijing, China), and the protein concentration was quantified using a BCA Protein Assay Kit (Nanjing Jiancheng Bioengineering Institute, Nanjing, China).

### 2.5. Oil Red O (ORO) Staining of Adipose Tissue

Fat accumulation in *C. elegans* was detected by ORO staining according to a previously described protocol [16]. The ORO stock solution was diluted with deionized water to 60% (*v*/*v*) and filtered through a 0.22 µM filter to remove impurities. Nematodes in different groups were completely washed with M9 buffer. Next, 600 µL of 40% isopropanol was used to fix nematodes. Subsequently, the samples were centrifuged at 560× *g* for 1 min to obtain *C. elegans*, which was stained with 600 µL of ORO solution for 2 h. After dyeing, the supernatant was removed by centrifugation, and excess floating color was removed by washing with M9 buffer. Cells were photographed under a microscope (Nikon Eclipse E100). The experiments were performed in triplicate. Ten nematodes in each experimental group were photographed. Images were analyzed using the ImageJ 1.52a version software (National Institutes of Health, Bethesda, MD, USA).

### 2.6. Measurement of Reactive Oxygen Species (ROS)

The assay was performed according to a previously described protocol with slight modifications [17]. Nematodes were collected 24 h after the intervention and completely washed with M9 buffer. The worms were transferred to 0.5 mL M9 buffer containing 5 μM H_2_DCFDA and preincubated in protection from light for 3 h at 20 °C. Then, 200 μL per well of M9 buffer with the probe and nematodes (1000 worms) was added to a 96-well plate, and the results were read using a fluorescence microplate reader (Thermo Scientific™ Multiskan™ FC, Waltham, MA, USA) at an excitation wavelength of 485 nm and an emission filter of 535 nm. The quantified ROS levels were expressed as an increase in fluorescence in 2 h.

### 2.7. Antioxidant Enzyme Assay

Catalase (CAT), glutathione peroxidase (GSH-Px), and superoxide dismutase (SOD) activities were assayed using commercially available kits (Solarbio Science and Technology Co., Ltd., Beijing, China) and were processed according to the manufacturer’s instructions. A BCA protein assay kit was used to quantify the protein concentrations in the homogenates of worms.

### 2.8. Free Fatty Acid Composition Analysis

Total free fatty acids were extracted and methylated as previously described [18]. Fatty acid methyl esters were then injected for gas chromatography/mass spectrometry analysis using an HP-5MS column (30 m × 0.25 mm × 0.25 µm) with helium as the carrier gas. The injector temperature was maintained at 250 °C. The initial temperature was 60 °C, which was increased to 150 °C at 15 °C/min, held for 3 min, increased from 5 °C/min to 195 °C for 5 min, increased from 6 °C/min to 230 °C for 3 min, and finally increased to 260 °C at 3 °C/min. The composition of fatty acids of *C. elegans* was identified by comparing the retention time and mass spectra with the commercial 37-component fatty acid methyl ester mixed standard. The desaturation index was calculated as the ratio of palmitoleic acid to palmitic acid or oleic acid to stearic acid [19].

### 2.9. Transcriptome Sequencing

Total RNA was extracted using TRIzol reagent at 4 °C, and the quality of the RNA was assessed using NanoDrop^TM^ 2000 (Thermo Fisher Scientific, MA, USA) and RNase-free agarose gel electrophoresis. Total RNA from each sample was subjected to Illumina ^NovaSeq^ 6000 (Shanghai Majorbio Biopharm Technology Co., Ltd.) (Shanghai, China). Single-end and paired-end RNA-seq libraries were prepared following Illumina protocols.

The reads obtained from the sequencing machines were subjected to the following processes: filtering of clean reads, alignment with ribosomal RNA, alignment with the reference genome, and quantification of gene abundance. Finally, RNA differential expression analysis was performed using the DESeq2 software between two different groups and by Edger between two samples. Genes with a false discovery rate (FDR) < 0.05 and an absolute fold change ≥ 2 were differentially expressed.

All differentially expressed genes were mapped to Gene Ontology (GO) terms in the Gene Ontology database (http://www.geneontology.org/, accessed on 3 March 2022), and Kyoto Encyclopedia of Genes and Genomes (KEGG) pathway enrichment analyses were subsequently performed to identify significant pathways. The calculated *p*-value was subjected to FDR correction, with FDR ≤ 0.05, as the threshold.

### 2.10. Quantitative Real-Time PCR

After 24 h of intervention, the nematodes in each group were collected and cleaned using M9 buffer. The main DEGs were validated using real-time quantitative reverse-transcription PCR. Real-time PCR was performed using a HiScript II Q RT SuperMix Kit (Enzyme Biotech Co., Ltd., Beijing, China), and Power SYBR Green PCR Master Mix (Enzyme Biotech Co., Ltd., Beijing, China) was used to perform cDNA synthesis and quantitative PCR according to the manufacturer’s protocols. Gene expression levels were calculated using the 2^−ΔΔCT^ method and were normalized to β-actin expression [20]. The primer sequences used for qPCR are listed in the Supplementary Material S1.

### 2.11. Data Analysis

Data are expressed as the mean ± standard deviation. Graphs were generated using Prism 8.0.1 (GraphPad Software, San Diego, CA, USA). The results were analyzed using IBM SPSS Statistics software (version 25.0; IBM, Chicago, IL, USA), followed by one-way analysis of variance (ANOVAs) with Duncan tests for post hoc comparisons among groups. Statistical significance was set at *p* < 0.05. Statistical analysis of variance between two groups was performed using the Student’s *t*-test. Differences were considered significant at *p* < 0.05 (*) and *p* < 0.01 levels (**). mRNA-seq analysis was performed using the online Majorbio Cloud Platform (www.majorbio.com, accessed on 1 December 2022).

## 3. Results

### 3.1. Healthy Lifespan of Nematodes Affected by P-AKK Under High Glucose Conditions

**Figure 1:**
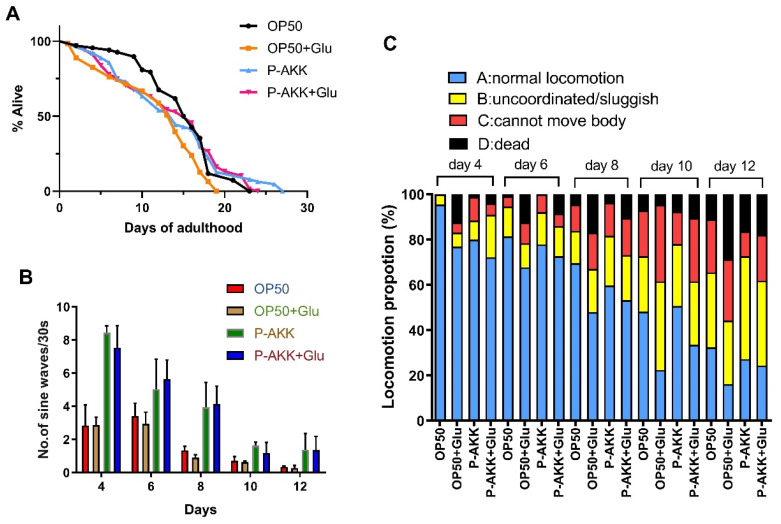
Effect of P-AKK on the healthy lifespan of nematodes under high glucose conditions. (**A**) Lifespan of nematodes (N = 3). (**B**) Nematode body bend frequency There were 10 worms in each group and the experiments were independently repeated three times. (**C**) Movement status of the nematodes. There were 10 worms in each group, and the experiments were independently repeated three times.

The healthy lifespan of nematodes includes the average lifespan, movement speed, and motility status. The results showed that the average lifespan of the OP50 + Glu group was 20.21% shorter than that of the OP50 group. However, the average lifespan of nematodes in the P-AKK group was similar to that of nematodes in the P-AKK + Glu group. However, the average life expectancy of the P-AKK + Glu group was 14.09% longer than that of the OP50 + Glu group. In addition, the longest lifespan of nematodes in the P-AKK + Glu group was 24 days, which was close to that of normally fed nematodes (Figure 1A, Table 1). Hence, the above results suggest that feeding on OP50 could not change the high glucose-induced shortening of the average lifespan of nematodes, but feeding on P-AKK helped to protect the normal lifespan of nematodes.

Motricity is an important indicator of a healthy lifespan, and the exercise speed gradually decreases with increasing nematode age. In all groups, the speed of nematode movement was negatively correlated with age, and physical exercise was slower. The proportion of spontaneous movement of nematodes fed a high-glucose diet was lower, but the proportion of sinusoidal movement of nematodes fed P-AKK was always greater than that of nematodes fed OP50 at different ages (Figure 1B,C). This shows that P-AKK helps to maintain the healthy lifespan of nematodes.

### 3.2. Effects of P-AKK on Biomarkers of Glucose and Lipid Metabolism in Nematodes Under High Glucose Conditions

Glucose is the main energy source and metabolic intermediate of animal cell metabolism; nevertheless, the long-term intake of large amounts of glucose harms cell and tissue function. Excessive carbohydrate intake activates several lipogenic enzymes in the body, leading to lipid metabolism disorders and obesity [21,22]. An imbalance in glucose metabolism leads to the long-term accumulation of methylglyoxal-derived AGEs and the production of ROS in the mitochondria, thus inducing cytotoxicity [23]. Therefore, the glucose, AGEs, and triglyceride contents were measured in nematodes fed a high-glucose diet.

**Figure 2:**
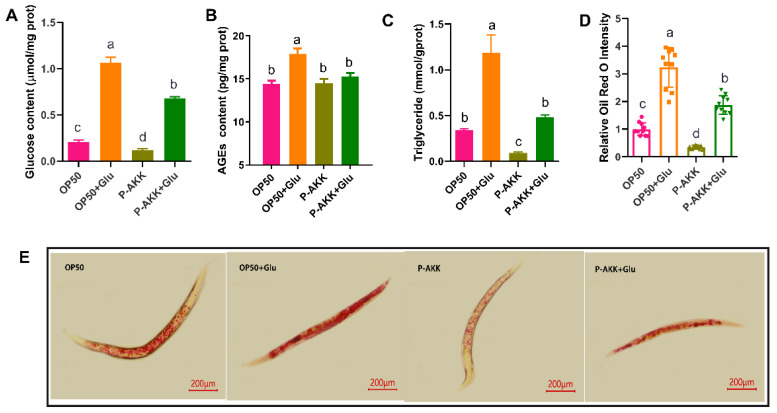
Effect of P-AKK dietary patterns on glucose and lipid accumulation in nematodes. (**A**) Glucose content in nematodes (N = 3); (**B**) AGE content in nematodes (N = 3); (**C**) triglyceride content in nematodes (N = 3); (**D**) quantification of fat content in nematodes stained with Oil Red O using ImageJ. There were 10 worms in each group, and the experiments were independently repeated three times. (**E**) Fat distribution in the nematodes stained with Oil Red O; the scale bar is 200 μm. The results are expressed as the mean ± SD, and *p*-values were calculated using Duncan’s test. Different lowercase letters above the bar graphs represent significant differences between groups (*p* < 0.05).

Compared to the OP50 group, the high-glucose diet significantly increased the intracellular glucose concentration, induced the accumulation of AGEs, and increased the triglyceride content in the OP50 + Glu group (*p* < 0.05). The obese phenotype of nematodes was induced by high glucose levels under the OP50 diet. However, the storage of glucose and TG in nematodes fed with P-AKK was lower than that in nematodes fed with OP50. Compared to the OP50 + Glu group, the accumulation of glucose, AGEs, and TG in the P-AKK + Glu group decreased by 36.07%, 14.59%, and 59.33%, respectively (*p* < 0.05) (Figure 2A–C). Moreover, using Oil Red O staining to visualize the lipid droplet distribution in nematodes, it was found that the lipid droplets in nematodes were distributed at a high density in a high-glucose environment, and the number of lipid droplets in P-AKK-fed nematodes was lower than that in nematodes fed OP50 (Figure 2E). These results are consistent with the fat quantification results of Oil Red O staining (Figure 2D).

### 3.3. P-AKK Changed the Level of Free Fatty Acids in Nematodes

The fatty acid composition of the nematodes was dominated by 16-, 18-, and 20-carbon chains. For example, palmitoleic acid (C16:1) and oleic acid (C18:1) are essential substrates for triglyceride biosynthesis [24]. Fatty acid composition is closely related to fat accumulation; therefore, changes in free fatty acids in nematodes were evaluated.

**Figure 3:**
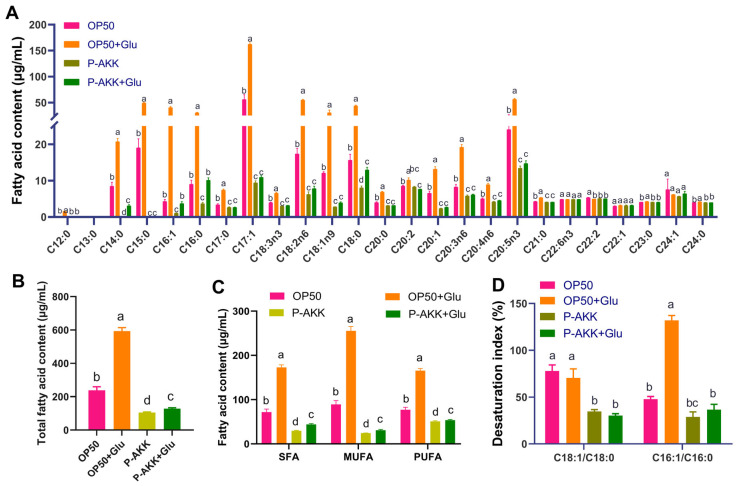
Effects of P-AKK on the free fatty acid composition of nematodes under high glucose conditions. (**A**) The free fatty acid content of nematodes (N = 3); (**B**) total free fatty acid content of nematodes (N = 3); (**C**) SFA, MUFA, and PUFA contents of nematodes (N = 3); (**D**) the desaturation index of nematodes (N = 3). The results are expressed as mean ± SD, and *p*-values were calculated using Duncan’s test. Significant differences between groups are indicated by different letters (*p* < 0.05).

As shown in Figure 3, compared to those in the OP50 group, the contents of SFAs, MUFAs, and PUFAs were significantly higher in the OP50 + Glu group, and the total fatty acid content was also significantly higher (*p* < 0.05). Among them, C16:1 and C18:1 were significantly increased in the OP50 + Glu group and were 8.37 and 1.52 times greater than those in the OP50 group, respectively (*p* < 0.05, Figure 3A). The desaturation index C16:1/C16:0 ratio in the OP50 + Glu group was 2.75-fold higher than that of the OP50 group (Figure 3D). High glucose levels increased the substrate concentration for TG synthesis and promoted the conversion of palmitic acid to palmitic acid, which was helpful for the synthesis and accumulation of TG. Compared with the OP50 diet, feeding P-AKK changed the composition of free fatty acids in nematodes. Even in a high-sugar diet, consuming P-AKK maintained the contents of SFA, MUFA, PUFA, and total free fatty acids in nematodes at a reduced level (Figure 3B,C). The desaturation indices C16:1/C16:0 and C18:1/C18:0 decreased significantly in the P-AKK + Glu group, by 72.18% and 55.93% (*p* < 0.05), respectively, compared with those in the OP50 + Glu group (Figure 3D).

### 3.4. Effects of P-AKK on the Intracellular ROS Level of Nematodes Under High Glucose Conditions

Adipose factors induce ROS production and oxidative stress, which causes lipid membrane oxidation and leads to the gradual accumulation of fat [25,26]. Thus, mitochondrial dysfunction directly affects the efficiency of glucose and lipid metabolism; however, restoring mitochondrial activity may be beneficial for preventing obesity [27].

**Figure 4:**
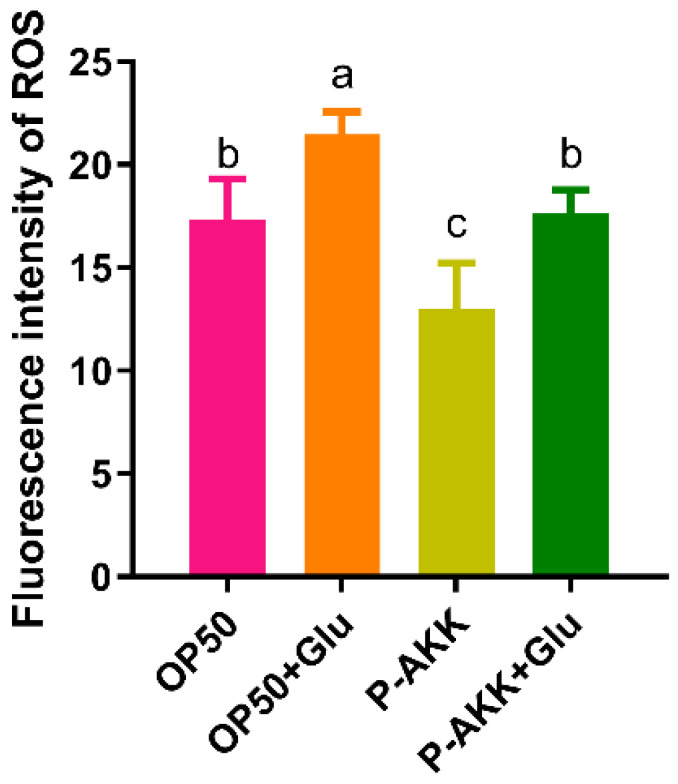
Effect of P-AKK on ROS levels in nematodes. The results are expressed as mean ± SD (N = 3,1,000 worms in each independent experiment), and *p*-values were calculated using Duncan’s test. Significant differences between groups are indicated by different letters (a–c) (*p* < 0.05).

Intracellular ROS levels were evaluated using the fluorescent probe, H_2_DCFDA. Based on OP50 dietary patterns, a high-glucose diet induces ROS accumulation in nematodes. However, when the nematode was fed P-AKK, the amount of ROS in the body was low, even under high glucose conditions, and the intracellular ROS level was consistent with the normal level. Additionally, compared with the OP50 + Glu group, the ROS level in the P-AKK + Glu group decreased by 17.67% (Figure 4). The results showed that P-AKK reduced intracellular ROS levels in nematodes fed a high-glucose diet.

### 3.5. Effects of P-AKK on the Antioxidant Activity of Nematodes Under High Glucose

**Figure 5:**
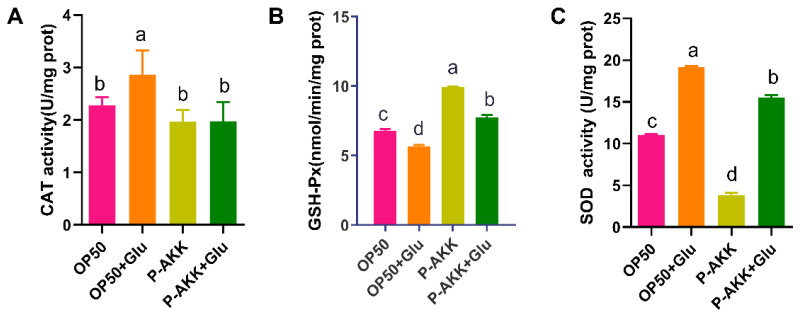
Levels of antioxidant enzymes in nematodes. (**A**) CAT activity level (N = 3); (**B**) GSH-PX activity level (N = 3); and (**C**) SOD activity level (N = 3). The results are expressed as the mean ± SD, and *p*-values were calculated by Duncan’s test. Significant differences between groups are expressed by different letters (*p* < 0.05).

Figure 5 results showed that compared with the OP50 group, the CAT activity significantly increased by 25.79%, and the SOD activity significantly increased by 72.75%, but the GSH-Px activity decreased in the OP50 + Glu group (*p* < 0.05). OP50 intake may induce oxidative stress and lead to a secondary increase in CAT activity in nematodes fed a high-glucose diet. However, the CAT activity of nematodes fed P-AKK did not change significantly, even under a high-glucose diet, which could not stimulate oxidative stress. Moreover, the CAT activity of nematodes in the P-AKK + Glu group was lower than that in the OP50 + Glu group, but the GSH-Px activity significantly increased (*p* < 0.05). Therefore, P-AKK may suppress oxidative stress by decreasing CAT activity and enhancing GSH-Px activity in worms.

### 3.6. Effects of P-AKK on Lipid Metabolism in Nematodes According to Gene Expression Profiles Under High Glucose Conditions

#### 3.6.1. Identification of DEGs

**Figure 6:**
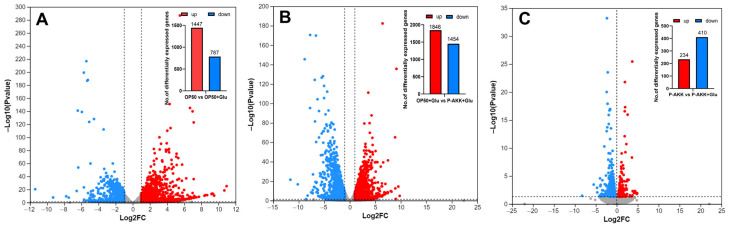
Scatter plot and statistical chart of differentially expressed genes in nematodes in different treatment groups. (**A**) OP50 vs. OP50 + Glu, (**B**) OP50 + Glu vs. P-AKK + Glu, (**C**)P-AKK vs. P-AKK + Glu. Note: The abscissa is the difference in the expression of the gene/transcript between the two samples; that is, the expression of the treatment sample was divided by the expression of the control sample, and the ordinate is the statistical test value of the difference in gene expression, namely, the *p*-value (N = 3).

Based on the phenotypic data from the above experiments, high-throughput sequencing was used for the systematic analysis of gene expression to further understand the physiological effects and mechanisms of different dietary patterns on nematodes. A rigorous comparison at adjusted *p* ≤ 0.05 and log2FC fold change ≥ 2 was performed to identify the number of DEGs for different groups (Figure 6). Through pairwise comparison, it was found that there were 2234 DEGs in the OP50 + Glu group compared with the OP50 group, of which 787 genes were downregulated and 1447 genes were upregulated. Compared to those in the OP50 + Glu group, there were 3300 DEGs in the P-AKK + Glu group, of which 1454 genes were downregulated and 1846 genes were upregulated. A total of 644 DEGs were obtained in the P-AKK + Glu group compared to the P-AKK group, of which 410 genes were downregulated and 234 genes were upregulated. These results indicated that feeding P-AKK significantly influenced the transcription of genes related to high glucose levels in nematodes.

Using iPath3.0 software (http://pathways.embl.de, accessed on 3 March 2022) to visually analyze the metabolic pathways associated with these DEGs, we determined which metabolic pathway was most affected. The results showed that the metabolic pathway was mainly involved in lipid and carbohydrate metabolism (Supplementary Material S2). This may be related to nematode feeding with high glucose levels, which induce a positive response to carbohydrate and lipid metabolism.

#### 3.6.2. Functional Prediction of DEGs Induced by P-AKK in the High-Glucose Diet

**Figure 7:**
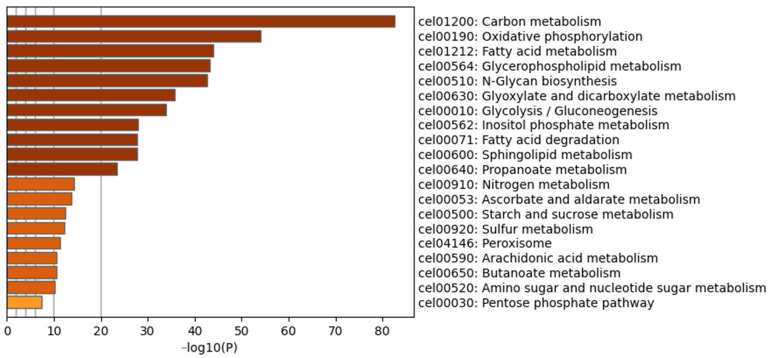
Histogram of the top 20 ranked GO terms of DEGs.

To explore the physiological regulatory effects of P-AKK on nematodes fed a high-glucose diet, pairwise comparisons were made between the P-AKK + Glu group and the OP50 + Glu group. The corresponding functional set information was obtained from the Gene Ontology (GO) database, and the importance of this functional set to DEGs was calculated. Figure 7 shows the top 20 ranked GO terms for the DEGs. Carbohydrate metabolism had the strongest degree of enrichment, followed by oxidative phosphorylation and fatty acid metabolism. These significantly enriched biological processes were closely related to glucose and lipid metabolism, indicating that P-AKK ingestion by nematodes significantly affected the biological processes of energy metabolism.

#### 3.6.3. KEGG Pathway Analysis

**Figure 8:**
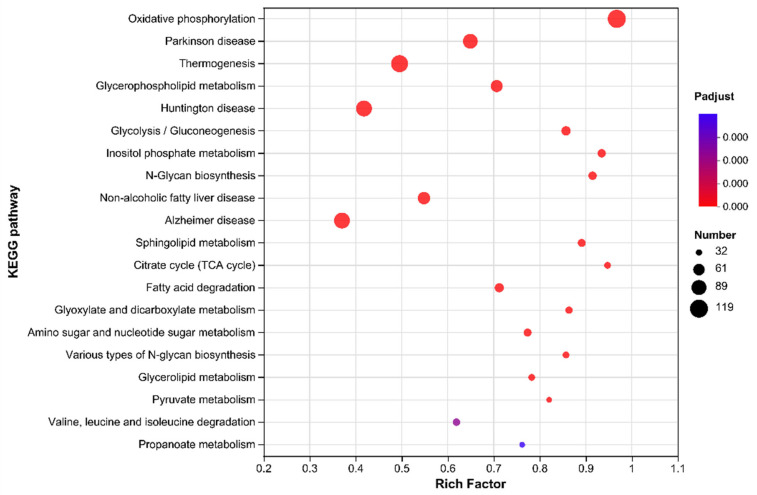
Bubble diagram of KEGG pathways.

Pathway analysis helps elucidate the biological functions of genes. By analyzing the KEGG pathways of the DEGs between the two groups, 266 typical pathways of significant enrichment were identified, of which 90 pathways showed the most significant difference (*p* < 0.01). Figure 8 shows the first 20 pathways of significant enrichment using a bubble chart. The results showed that the metabolic pathway of oxidative phosphorylation had the highest enrichment degree (enrichment factor of 0.97), followed by the TCA cycle pathway (enrichment factor of 0.95). In addition, the pathways of glycerol phospholipid metabolism, glycolysis/gluconeogenesis, fatty acid degradation, glyoxylic acid and dicarboxylic acid metabolism, and pyruvate metabolism were also significantly enriched, and these pathways were directly related to glucose and lipid metabolism. Among these pathways, P-AKK upregulated the glycolysis/gluconeogenesis pathway, glyoxylate and dicarboxylate metabolism pathway, and fatty acid degradation pathway, and downregulated the citrate cycle and fatty acid biosynthesis (Supplementary Material S3). Consequently, it can be speculated that one underlying mechanism by which P-AKK improves high-glucose-induced glycolipid metabolism is the regulation of dietary energy intake.

### 3.7. qPCR Validation of Differentially Expressed Genes

**Figure 9:**
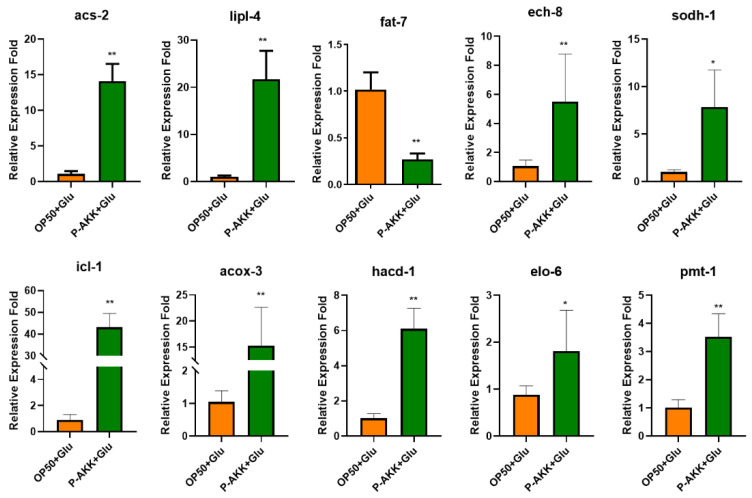
Effects of P-AKK on DEGs in *C. elegans* fed a high-glucose diet (n = 3). All data are expressed as mean ± standard deviation relative to the OP50 + Glu group. * *p* < 0.05, ** *p* < 0.01.

To verify the changes in key genes regulated by P-AKK, several DEGs, including *pmt-1*, *lipl-4*, *sodh-1*, *ech-8*, *icl-1*, *elo-6*, *fat-7*, *acox-3*, *hacd-1*, and *acs-2*, were selected for qPCR verification. These detection results were consistent with those of RNA-seq. Compared with that in OP50 + Glu, *fat-7* gene expression significantly decreased by 74.79%, *acs-2* gene expression significantly increased by 12.08 times, *lipl-4* gene expression increased by 20.05-fold, *icl-1* gene expression increased by 46.18-fold, and *acox-3* gene expression increased by 13.59-fold (*p* < 0.01, Figure 9). These results were in accordance with those of the phenotype experiments.

## 4. Discussion

In the present study, nematodes fed P-AKK exhibited better physiological phenotypes. The P-AKK diet improved the lifespan and mobility of *C. elegans* induced by a high-glucose diet. In addition, P-AKK intake regulated intracellular glucose concentration and AGE accumulation in a high-glucose diet, especially by decreasing triglyceride content and fat distribution density. Using RNA-seq to determine the whole gene expression profile of *C. elegans*, we found that feeding nematodes P-AKK significantly affected lipid metabolism by regulating the glucose metabolism pathway. Unlike our prior work demonstrating the nutritional adequacy of P-AKK under a standard diet [11], the current study establishes its therapeutic potential in counteracting glucose-induced metabolic dysregulation.

A diet of P-AKK is more beneficial to the healthy lifespan of *C. elegans*. In *C. elegans*, high glucose levels play a harmful role by increasing ROS production, TG accumulation, and AGE formation [28]. The lifespan of nematodes is shortened, and the level of oxidative stress increases after exposure to high concentrations of glucose [29]. In this study, the lifespan of nematodes fed P-AKK did not decrease in response to high glucose, and P-AKK protected them from a healthy lifespan (including motoricity) in a high-glucose environment. Furthermore, P-AKK reduced ROS levels and cytotoxicity by enhancing the activity of GSH-PX. ROS can easily cause DNA damage, and the accumulation of damage accelerates aging and affects healthy lifespan. Nevertheless, P-AKK can enhance the motoricity of nematodes to increase metabolic flux and the activity of antioxidant enzymes to eliminate excessive ROS and reduce the damage caused by a high-glucose diet. This explanation is supported by Xiong et al. [30]. It functions similar to oral *Pediococcus acidilactici* in that it decreases ROS levels, increases nematode longevity, and indirectly regulates lipid metabolism in vivo [31]. As a result, P-AKK consumption in nematodes benefits their healthy lifespan by preventing mitochondrial dysfunction and enhancing cell metabolic flux.

The P-AKK diet improved the glucose and lipid metabolism in nematodes. Excessive dietary sugar has been reported to weaken the insulin sensitivity of cells, resulting in a decrease in glucose uptake and an increase in glucose concentration in systemic circulation, whereas hyperglycemia induces protein glycosylation and causes cytotoxicity, which has a negative effect on protein, tissue, and organ function [32]. However, the intake of P-AKK helps nematodes to inhibit the stress toxicity of glucose and the cytotoxicity of AGEs under a high-glucose diet. This may be related to a decrease in glucose absorption by P-AKK [33]. Excessive glucose intake may upset the balance of fatty acids in the body, resulting in metabolic and cellular changes [34]. In this study, high glucose induced an overall increase in free fatty acids in nematodes and promoted the transformation of palmitic acid to palmitic acid, resulting in the accumulation of TG. However, the dietary intake of P-AKK could improve this high-glucose response, inhibit fatty acid desaturation, and reduce nematode fat accumulation. The quantitative results of Oil Red O staining also supported this conclusion.

According to the results of RNA-seq analysis, P-AKK not only has a regulatory effect on the carbohydrate metabolism pathway but also significantly affects the transcription of genes involved in lipid metabolism. P-AKK upregulated pathways involved in fatty acid degradation and glyoxylate and dicarboxylate metabolism and downregulated the de novo fat synthesis pathway. Therefore, it was speculated that P-AKK enhanced cell metabolic activity and significantly regulated lipid metabolism. P-AKK decreased the level of ROS and upregulated the fatty acid degradation pathway, which caused nematodes to maintain very low-fat storage, which may be related to fatty acid β-oxidation. In this study, nematode feeding on P-AKK significantly promoted the transcription of *lipl-4* and *acs-2* genes in the biological process of fatty acid β-oxidation under high-glucose conditions. *Lipl-4* encodes lipase, and increased lipase expression indicates increased lipid hydrolysis. In addition, LIPL-4 lipases mediate specific lysosomal signals and induce redox reactions in nematode mitochondria, thereby reducing lipid storage [35]. *Acs-2,* which encodes a fatty acid CoA synthetase, is the key target that induces fatty acid β-oxidation, and its upregulated expression could indicate enhanced fatty acid β-oxidation. Therefore, the synergistic effect of *lipl-4* and *acs-2* contributes to β-oxidation of fatty acids in nematodes. Additionally, the overexpression of *acs-2* causes *C. elegans* to be in a low-fat state [36]. Moreover, P-AKK significantly upregulated genes downstream of fatty acid β-oxidation, such as *ech-8* and *sodh-1*, indicating that P-AKK promotes the fatty acid β-oxidation pathway. Additionally, P-AKK significantly downregulated *fat-7* expression. *Fat-7* encodes stearoyl-CoA desaturase (SCD), which is the key enzyme for the conversion of palmitic acid and stearic acid to palmitic acid and oleic acid, respectively [37]. Glucose generates palmitic acid during lipid de novo synthesis and then upregulates SCD, causing palmitic acid to generate palmitoleic acid. This may be one of the mechanisms by which carbohydrate intake leads to an increase in palmitoleic acid in the body [38]. Therefore, we speculate that to counteract the excessive fat storage caused by a high-glucose diet, P-AKK may improve the health of nematodes by enhancing their ability to exercise, promoting β-oxidation of fatty acids, and maintaining a low-energy state. Another assumption is that there is a difference in the energy metabolism between OP50 and inactivated AKK because of the metabolic cycle capacity of living bacteria. The eating pattern of P-AKK is similar to that of a low-calorie diet, in which foods contain fewer calories while ensuring adequate dietary intake. Overall, the intake of P-AKK by nematodes is a healthier dietary mode that helps nematodes to stay low in fat. However, these speculations require further investigation.

## 5. Conclusions

In summary, the administration of P-AKK had a positive effect on the body phenotypes of *C. elegans* under high glucose conditions. Consumption of P-AKK enhanced motor activity, which decreased with age and protected the healthy lifespan of nematodes fed a high-glucose diet. P-AKK has strong metabolic activity and decreases the accumulation of glucose, AGEs, and TG in vivo. These effects result from a combination of key enzymes involved in fatty acid synthesis and oxidation processes. Among these, acyl-CoA synthetase and stearoyl-CoA desaturase play significant roles. Thus, P-AKK may be a potential postbiotic that can be used as a dietary supplement to change the dietary structure and improve fatty acid metabolism.

## Figures and Tables

**Table 1:** Lifespans of nematodes subjected to different types of bacterial treatments.

Group	Number of Worms	Mean Lifespan (Days)	Maximum Lifespan (Days)	Median (Days)
OP50	63	14.94 ± 0.58 a	23	15.5
OP50 + Glu	68	11.92 ± 0.69 b	19	14.0
P-AKK	68	13.78 ± 0.81 ab	27	14.0
P-AKK + Glu	63	13.60 ± 0.78 ab	24	16.0

Note: Means with different letters in the same column are significantly different (*p* < 0.05).

## Data Availability

The authors confirm that the data supporting the findings of this study are available in the article and its Supplementary Materials.

## Supplementary Materials

The following supporting information can be downloaded at: https://www.mdpi.com/article/10.3390/nu17060976/s1, Supplementary Material S1 Primer sequences. Supplementary Material S2. Metabolic pathway map of the nematodes. Supplementary Material S3. P-AKK + Glu and OP50 + Glu groups pairwise compared differential genes in the glucose and lipid metabolism pathways. All authors have read and agreed to the published version of the manuscript.
