# Supplementary material for: The Positive Effect of Akkermansia muciniphila postbiotics on the Glycolipid Metabolism of Caenorhabditis elegans Induced by High-Glucose Diet
Source: Nutrients. 2025 Mar 11;17(6):976. doi: 10.3390/nu17060976 (PMC11945073; doi:10.3390/nu17060976)
Supplement: Supplementary file 1 [file nutrients-17-00976-s001.zip › Supplementary material S1.pdf]

Supplementary Material S1 Primer sequences

Genes	Forward primer sequence (5′ to 3′)	Reverse primer sequence (5′ to 3′)
β-actin	GCCGGAGACGACGCTCCACGCG	GCCTCGTCTCCGACGTACGAGTC
lipl-4	ATGGCCGAGAAGTTCCTACATCGT	GGTGAATTGGCGACCCAATCGAAA
acs-2	GCAGCCTCGCTCTACACTCT	GACTCCTGCAAATGCACATGC
fat-7	CAACAGCGCTGCTCACTATT	CACCAACGGCTACAACTGTG
pmt-1	GCTTCTCCGTGCCATTCGCTAC	AGTGGACTTGACGCCAGTTGTTG
sodh-1	AGCCACTCTGCCACCACATCC	CAGCGGCGAGATTGGTATCCTTG
ech-8	TCATCAATTGGCGGCTCAGGTAAC	TCTACAGAGTGCCCACGGATACG
icl-1	GACTACGAGGCTGGAAGAACGATTG	ATGGAGCATATTGGATGGCACGATC
elo-6	TCCACCAATCCACCCACTTAAAACC	AAATAGCAAGACCCGCATTCCAGAG
acox-3	GCTAATTGGCTTGAAAGGGTTGTGC	GACATTGGAGAATCAGACGGACGAG
hacd-1	AGGTCTGTCTTCAGGCAGGCTATC	CCATCGGAACGTCGGTCTTCTTC
