# Supplementary material for: The Positive Effect of Akkermansia muciniphila postbiotics on the Glycolipid Metabolism of Caenorhabditis elegans Induced by High-Glucose Diet
Source: Nutrients. 2025 Mar 11;17(6):976. doi: 10.3390/nu17060976 (PMC11945073; doi:10.3390/nu17060976)
Supplement: Supplementary file 1 [file nutrients-17-00976-s001.zip › Supplementary material S2 .pdf]

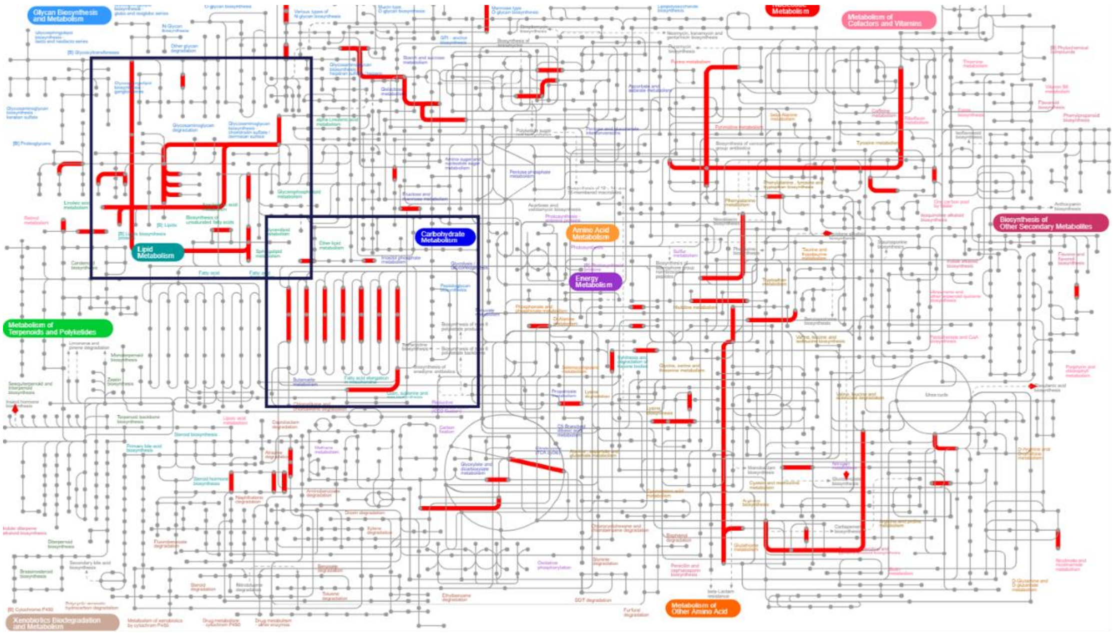

Supplementary Material S2. The metabolic pathway map of nematodes.

Note: Nodes represent different compounds and boundaries represent different enzymatic reactions.
