# Supplementary material for: The Positive Effect of Akkermansia muciniphila postbiotics on the Glycolipid Metabolism of Caenorhabditis elegans Induced by High-Glucose Diet
Source: Nutrients. 2025 Mar 11;17(6):976. doi: 10.3390/nu17060976 (PMC11945073; doi:10.3390/nu17060976)
Supplement: Supplementary file 1 [file nutrients-17-00976-s001.zip › Supplementary material S3.pdf]

Supplementary Material S3. P-AKK+Glu group and OP50+Glu group pairwise comparison of differential genes in glucose and lipid metabolism pathway

KEGG pathway	Gene name	Gene ID	Function	Fold change	State
Glycerophospholipid metabolism	*gpdh-1*	WBGene00009824	Glycerol-3-phosphate dehydrogenase	0.195	down
	acl-1	WBGene00010339	1-acyl-sn-glycerol-3-phosphate acyltransferase	0.379	down
	plpp-1.2	WBGene00020895	Phospholipid phosphatase homolog	0.430	up
	pmt-1	WBGene00022781	Phosphoethanolamine MethylTransferase	2.803	up
	Y69A2Al.2	WBGene00044468	Phospholipase	0.274	down
	lipl-1	WBGene00010062	Lipase	4.028	up
	lipl-3	WBGene00020016	Lipase	472.676	up
	lipl-4	WBGene00019376	Lipase	10.391	up
	lipl-7	WBGene00008510	Lipase	2.592	up
Glycolysis/Gluconeogenesis	*sodh-1*	WBGene00010790	Alcohol dehydrogenase 1	4.812	up
Citrate cycle (TCA cycle)	*acly-1*	WBGene00016995	ATP-citrate synthase	0.498	down
	D2023.8	WBGene00050970	Pyruvate carboxylase	0.408	down
Fatty acid degradation	*ech-8*	WBGene00001157	Enoyl-CoA Hydratase	2.085	up
	B0272.4	WBGene00007130	Catalytic activity	2.105	up
	acox-1.5	WBGene00008167	Peroxisomal acyl-coenzyme A oxidase	0.23	down
	sodh-1	WBGene00010790	Alcohol dehydrogenase 1	4.812	up
	acdh-2	WBGene00015894	Acyl CoA DeHydrogenase	0.233	down
	acs-1	WBGene00018488	Fatty Acid CoA Synthetase	0.253	down
Glyoxylate and dicarboxylate metabolism	*ctl-2*	WBGene00000831	Peroxisomal catalase	2.068	up
	icl-1	WBGene00001564	Glyoxylate cycle protein Isocitrate lyase Malate synthase	3.549	up

	gln-5	WBGene00001606	GLutamiNe synthetase	0.479	down
	gln-6	WBGene00001607	GLutamiNe synthetase	0.457	down
	C05D11.5	WBGene00015483	Hydroxypyruvate isomerase	0.246	down
	pgph-3	WBGene00016892	PhosphoGlycolate Phosphatase	0.251	down
	pgph-2	WBGene00018424	PhosphoGlycolate Phosphatase	0.258	down
	T02G5.7	WBGene00020166	Transferase	3.387	up
Glycerolipid metabolism	*dgat-2*	WBGene00010296	Acyl-CoA:DiacylGlycerol AcylTransferase	0.255	down
	acl-1	WBGene00010339	1-acyl-sn-glycerol-3-phosphate acyltransferase	0.379	down
	plpp-1.2	WBGene00020895	Phospholipid phosphatase homolog	0.430	up
Pyruvate metabolism	*T02G.7*	WBGene00020166	Transferase	3.387	up
	D2023.8	WBGene00050970	Pyruvate carboxylase	0.408	down
Biosynthesis of unsaturated fatty acids	*dhs-27*	WBGene00000990	Oxidoreductase	3.037	up
	elo-6	WBGene00001244	Elongation of very long chain fatty acids protein	3.574	up
	fat-6	WBGene00001398	Delta(9)-fatty-acid desaturase	2.264	up
	fat-7	WBGene00001399	Delta(9)-fatty-acid desaturase	0.205	down
	acox-1.5	WBGene00008167	Peroxisomal acyl-coenzyme A oxidase	0.23	down
	D2021.4	WBGene00017048	Hydrolase	2.081	up
	maoc-1	WBGene00017123	MAO-C-like dehydratase domain	2.069	up
	acox-3	WBGene00019060	Peroxisomal acyl-coenzyme A oxidase	2.254	up
	K05B2.4	WBGene00019404	Acyl-CoA hydrolase	0.318	down
Fatty acid biosynthesis	*dhs-27*	WBGene00000990	Oxidoreductase	3.037	up
	ech-8	WBGene00001157	Enoyl-CoA Hydratase	2.085	up
	elo-6	WBGene00001244	Elongation of very long chain fatty acids protein	3.574	up
	Y48A6B.9	WBGene00012970	Enoyl-[acyl-carrier-protein] reductase	2.054	up

	acs-1	WBGene00018488	Fatty Acid CoA Synthetase	0.253	down
	K05B2.4	WBGene00019404	Acyl-CoA hydrolase	0.318	down
	hacd-1	WBGene00019978	Hydroxy-Acyl-CoA Dehydrogenase	4.17	up
	acs-2	WBGene00009221	Fatty Acid CoA Synthetase	2.852	up
